# Differences in Angular Photogrammetric Soft-Tissue Facial Characteristics among Parents and Their Offspring

**DOI:** 10.3390/medicina55050197

**Published:** 2019-05-23

**Authors:** Gordana Lj. Filipović, Nikola M. Stojanović, Ivan D. Jovanović, Pavle J. Randjelović, Ivan R. Ilić, Nadica S. Djordjević, Niko S. Radulović

**Affiliations:** 1 Department of Orthodontics, Dental Clinic, Faculty of Medicine, University of Niš, Zorana Đinđića 81, 18000 Niš, Serbia; filipovic.dr.gordana@gmail.com; 2 Faculty of Medicine, University of Niš, Zorana Đinđića 81, 18000 Niš, Serbia; 3 Department of Anatomy, Faculty of Medicine, University of Niš, Zorana Đinđića 81, 18000 Niš, Serbia; ivanjov@sbb.rs; 4 Department of Physiology, Faculty of Medicine, University of Niš, Zorana Đinđića 81, 18000 Niš, Serbia; pavleus@gmail.com; 5 Institute of Pathology, Faculty of Medicine, University of Niš, Zorana Đinđića 81, 18000 Niš, Serbia; ilicko81@gmail.com; 6 Dental Clinic, Faculty of Medicine, University of Priština Kosovska Mitrovica, Anri Dinana bb, 38220 Kosovska Mitrovica, Serbia; nadicadj@yahoo.com; 7 Department of Chemistry, Faculty of Sciences and Mathematics, University of Niš, Višegradska 33, 18000 Niš, Serbia; nikoradulovic@yahoo.com

**Keywords:** photogrammetry, Serbian population, angular measurements, parents, offspring

## Abstract

*Background and objectives:* The objective of this study was to determine if the angular photogrammetric analysis of soft-tissue characteristics can determine similarities between parents and their offspring in the Serbian population. *Materials and Methods:* A total of 15 families (52 participants) met the participation criteria of this study and their facial profile images were analyzed using the ImageJ software. Subjects were divided into groups of mothers and fathers and four groups of children (divided according to their age and gender). In total, twelve angular measurements were made on the standardized digital images of the profiles of the participants and the obtained data were compared using one-way ANOVA. *Results:* The obtained results showed that there were statistically significant differences in the values of the nasal and cervicomental angles, as well as the angle of the total facial convexity, between the group of fathers, on one side, and groups of male/female children, on the other. *Conclusions:* This work represents the first photogrammetric analysis of facial soft-tissue characteristics of children and adults in the Serbian population. The data suggest that there are much more similarities between the facial soft-tissue angles of fathers and their male offspring. Furthermore, mothers tend to have statistically insignificant differences in angle sizes, compared to both male and female offspring.

## 1. Introduction

Soft-tissue analysis using appropriate images of human faces (i.e., photogrammetry), has been used for a few decades to evaluate both developmental and age-related changes, as well as population variables in humans [1,2]. This method has primarily been utilized in orthodontics, genetics, maxillofacial and plastic surgery for diagnosis, treatment planning, and post-operative assessment [3,4,5,6]. Additionally, facial morphology is a useful tool for other scientific disciplines, such as anthropology, forensic science, sociology, psychology, and so on, with a common goal of investigating factors that affect facial morphology in different populations/states [7,8,9].

Studies have shown that the proportions and form of the face are influenced by genetics and environmental factors [10,11,12]. Genetic background plays an important role in the craniofacial structure and growth, and evidence for such claims can be found in studies conducted between twins and siblings [13,14,15]. Thus, these data can be considered useful in predicting craniofacial characteristics and could give important clues about parent-child heritability. However, the fact remains that there are few studies that determine the similarities between the parents and their offspring using facial morphology [16]. Researchers have introduced various facial landmarks and angles associated with this type of analysis, describing a new, different aspect of facial soft-tissue [17].

Growth and orthodontic treatment are known to be closely related to changes in the facial contours. Growth changes occur in different magnitudes, affecting all facial parts at some point [18]. During growth, due to chin protrusion, changes in the convexity of the skeletal profile, which are not followed by a consequential soft-tissue alteration, occur. Furthermore, significant changes in the nose and lip areas should be expected during growth [19]. These unequivocal changes in the facial tissue structure point to different mechanisms of facial growth and change [18]. It has been found that the thickening of different facial structures is delayed in boys, compared to girls, but growth is known to be more pronounced in boys [18]. 

The importance of soft-tissue characteristics is emphasized and applied in numerous fields of both medicine and orthodontics [3]. However, our work aims to evaluate a possible additional application of angular photogrammetric analysis of soft-tissue characteristics for the determination of similarities between parents and their offspring, for the first time in the Serbian population.

## 2. Materials and Methods

### 2.1. Subjects

Before the commencement of the study, each volunteer gave informed consent as to the purpose and nature of the study. All work has been performed in accordance with the Declaration of Helsinki and was approved, on the 10th of March 2016, by the Faculty Ethical committee (No. 12-2307-2/14). Our subjects were Caucasian parents (aged 38 to 56) and their children (from 15 to 21 years (y.) old), from Serbia, with no facial deformities.

All volunteers were recruited using a questioner and, after their initial involvement, the selected study group met the following criteria: Serbians with Serbian grandparents; all teeth present, apart from the third molars in some cases (children 17 years old and younger); good facial symmetry; and without significant medical history (no history of trauma, orthodontic or prosthodontic treatment, no maxillofacial or plastic surgery, and had Class I dentoskeletal relationships (ideal overjet and overbite)). Body mass index (BMI) was not taken into account during the collection and analysis of results. In total, 15 families (with a total of 52 participants) met the participation criteria of this study and were further analyzed. The study was conducted on a group coming from southeastern Serbia, in the municipal area of Niš. Children, as subjects, were divided into groups of males and females, and each group was further divided into two groups; those aged 15–17 and 18–21 y. As the Serbian ethical committee does not allow radiographic exposure of patients for the purposes of investigation, standardized facial photographs were used.

### 2.2. Camera Characteristics and Image Capturing

The camera (Canon EOS 650D) was positioned on a tripod (90 cm from ground level), the height of which was adjusted so that the optical axis of the camera lens remained horizontal and the film plane vertical. A leveling device (which is a part of a tripod) was used to ensure that the camera was in the correct horizontal position during the capture of the photographs. Additionally, the height of the tripod was adjusted for each subject (sitting position) when the photographs were taken. The objective lens was Sigma (Kawasaki, Japan) EX 105 mm f/2.8 DG Macro, and the shutter speed was set to 1/125 per second, with a fully opened diaphragm.

All subjects were placed in the same sitting position (2 m from the camera), in front of a calibration scale, turning their face/body profile to the camera, while their heads were in normal head position (NHP). Behind the subject, the same 30 cm-long vertical calibration scale was placed, where both centimeters and millimeters could be distinguished. This scale allowed calibration of the digital image to life-size dimensions during the measurement of photographs. The NHP position was achieved by carefully instructing the subjects to relax their bodies, put their arms close to their body, relax their lips, and watch their own eyes in the mirror placed in front of them. Subjects were also asked to remove their eyewear, all hair from the forehead, and the remaining exposed side of the face behind the ears.

### 2.3. Photogrammetric Measurement

**Figure 1:**
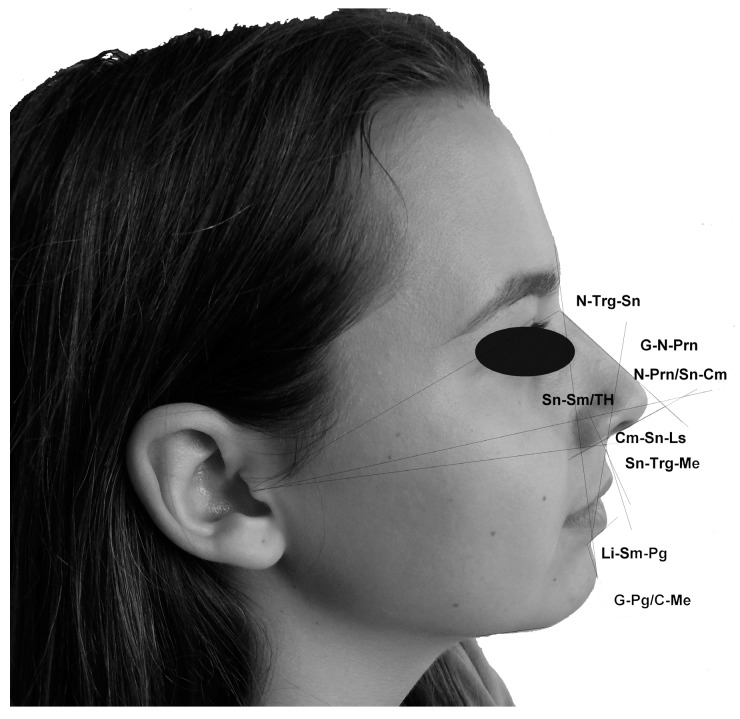
Angles measured included: Nasofrontal angle (G–N–Prn), vertical nasal angle (N–Prn/TV), nasolabial angle (Cm–Sn–Ls), mentolabial angle (Li–Sm–Pg), nasal angle (Cm-Sn/N–Prn), nasal dorsum angle (N–Mn–Prn), cervicomental angle (C-Me/G-Pg), middle facial third angle (N–Trg–Sn), lower facial third angle (Sn–Trg–Me), and angle of the head position (Sn–Sm/TH).

**Figure 2:**
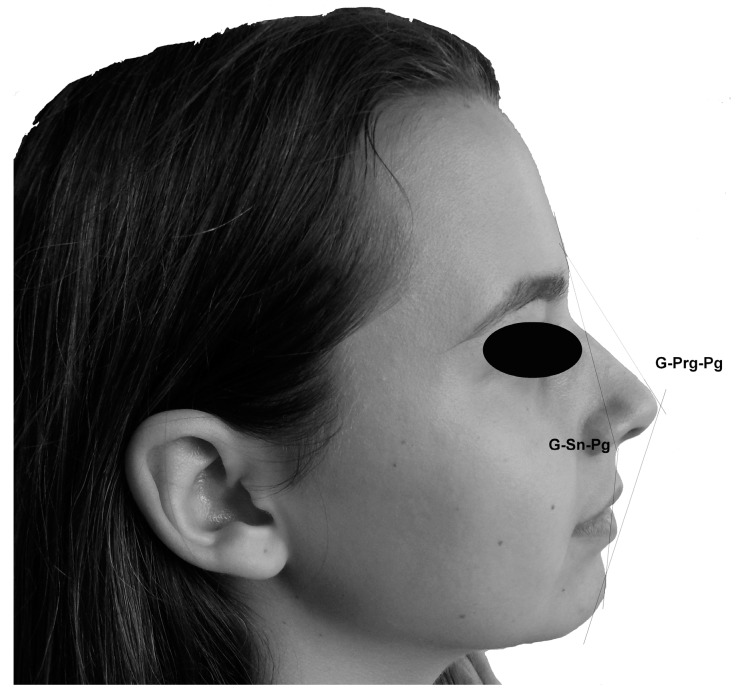
Angles corresponding to the facial convexity: The facial profile angle (convexity) (G–Sn–Pg) and the angle of the total facial convexity (G–Prn–Pg).

The obtained images of human faces in profile position were analyzed using the ImageJ software (http://rsb.info.nih.gov/ij/). Before any measurement, each image was calibrated using the ruler which was placed next to the face of the volunteer during the image capture. Details concerning the facial landmarks, which were used for the selected angular measurements, are shown in Table 1 and Figure 1 and Figure 2 and represent previously-established parameters [17]. In total, twelve angular measurements were made on the standardized digital images of the human profiles (Figure 1 and Figure 2). All measurements were performed by one researcher on black and white images.

### 2.4. Statistical Analysis

Comparisons between the groups of fathers/mothers and the four groups of their offspring were done using one-way ANOVA followed by Tukey’s post-hoc test, in order to estimate additional differences between the experimental groups (SPSS version 16). Probability values (*p*) less than 0.05 were considered to be statistically significant.

## 3. Results

This study included a total of 30 adults (15 males and 15 females) and 22 children, aged from 15 to 21, which were further divided (according to gender and age intervals) into 4 groups. Table 2 presents the measured (angle) parameters of parents and their offspring, as well as the results of the statistical analyses (ANOVA and Tukey’s post-hoc test). Only four out of twelve measured angles were significantly different, as revealed by ANOVA, between the parents and their children (Table 2). 

On the other hand, there were several parameters (measured angles) where the *p* values for the compared groups were almost 0.05 and, possibly, an increased number of subjects would alter the outcome of the statistical analyses. Tukey’s post-hoc test revealed less statistically significant differences than the previous analysis but gave better insight into the differences within the groups. From this analysis, we can see that there were statistically significant differences in the values of the nasal (Cm-Sn/N-Prn) and cervicomental angles (C-Me/G-Pg) and the angle of the total facial convexity (G-Prn-Pg) between the groups of fathers, on one side, and the groups of male children aged 15–17, and female children aged 15–17 and 18–21, on the other (for details, refer to Table 2). In the case of the group of mothers, there were no statistically significant differences found with the groups of children after Tukey’s post-hoc test was applied. This disappearance of statistical difference between groups of mothers and children could be explained by the small number of included subjects.

## 4. Discussion

We discuss our findings mainly with respect to growth: Heritage plays a big role, but the phenotype of the children is the result of a combination of genotypes and other factors. As mentioned in the introductory section, the nose structures undergo changes during growth and progress in the forward direction. Additionally, the nose soft-tissue has the greatest increase in size, compared to the lip and chin [19]. The analyzed nose soft-tissue describing angles included the nasal, vertical nasal, and nasal dorsum angles. In the case of the nasal angle, a statistical difference was found between the groups of fathers and female children 15–17 y. (Table 2). Interestingly, the nasal angle (tip of the nose) has been found to differ among the sexes, being somewhat larger in females than in males, by different authors [17]. This angle was found to reach adult values in girls at around 6 to 7 years, while in young boys nearly 86% reached adult values [20,21]. Such data are not supported by the results of our study, since the only differences that were found existed between fathers and younger daughters (Table 2). Data from the meta-analysis [22] suggests that, in the white (Caucasian) population, the values of the nasal angle are from 70.1° to 81.8° (mean 75.7°). The values outside this range were those obtained from the groups of older males and younger female children (Table 2).

The cervicomental angle is believed to be the hallmark of youth, together with other facial landmarks, and it varies in the range of 90–100°. With aging, this angle tends to become less acute (>100°) due to the loss of dermal elasticity, attenuation of platysma, increase in fatty tissues, and so on [23]. Thus, the results of our study (i.e., the presence of statistically significant differences between the groups of fathers and male/female children aged 15–17) were somewhat expected (Table 2). In slightly older children and the group of mothers, this angle tends to be similar (≈100°, Table 2). In Turkish and Iranian populations, this angle was found to be statistically different between the genders, aged 19–25 [24,25]. The values of these sex-related cervicomental angle differences, although pronounced, differed among the mentioned populations, in an interval from 76–101° [24,25]; whereas the values of adults (the groups of mothers and fathers) in our study were slightly higher (Table 2).

The lack of statistically significant differences (after the post-hoc analysis) in the facial profile angle (G-Sn-Pg) between the groups of mothers and the groups of children is in good agreement with the known fact that this angle remains relatively constant during growth [3]. Thus, one should not expect family/gender-related changes to occur in the value of this angle.

The angle of the total facial convexity was found to be statistically different between younger male children and mothers/fathers; however, after the post-hoc analysis, this difference was only visible between the groups of younger male children and fathers (Table 2). According to the meta-analysis by Wen et al. [22], this angle in the Caucasian population ranges from 138.2–145.4° (with mean 141.5°). The values obtained herein, for each of the study groups, were mostly accommodated within this range, except for the group of male children 15–17 y. (Table 2). Here, again, the sexual dimorphism of this angle is debatable; there are groups of authors claiming both the presence or absence of differences between males and females [1,17,26,27]. The reported angles of the total facial convexity were similar or somewhat smaller than those measured in our study (Table 2). The measured nasolabial angle (Cm-Sn-Ls) was previously found to have a large SD (>7°), in several studies which included subjects from all over the world (USA, Croatia, and Saudi Arabia) [1,2,26,28]. This angle was also demonstrated to statistically significantly differ between males and females at the age of 6 and 7 and in those aged from 25–29 [1,29,30]. Such large SDs and gender differences (or, as a matter of fact, any kind of difference between the analyzed groups) were not found in our study. These large SDs can possibly be explained by the fact that two out of three studies reporting this [2,28] used radiographs for the analysis. However, the explanation of the large SDs for the angle in question, in studies that used images for the angle measurements, could be interpreted as a high angle variability [1,26]. According to Wen et al. [22], the value range for this angle in the Caucasian population should be between 94.0–105.8°, with a mean value of 100.1°. The values in our study were much higher than those suggested by Wen et al. [22], and the values for this angle in children were more similar to the ones found in our study [29].

The mentolabial angle (Li-Sm-Pg), with expected values between 124.3–133.3° [22], did not differ in its value among the study groups (Table 2; *p* > 0.05). Again, as in the case of the nasal angle, the groups of older males and younger female children had values of the mentolabial angle below and above the mentioned range of values, respectively; but with a high SE (Table 2). Such a high SE can be expected for the mean values of this angle, as reported previously, and could be associated with the Class II profile cases [1]; which correspond, according to the facial profile angle, to the groups of older males and younger female children. In the groups of older males and females (fathers and mothers), the values for this angle were in the expected range and could be correlated with the values found by other researchers [26,31].

The values of the nasofrontal angle measured in our study groups were above (Table 2) the suggested range of values for the Caucasian population (from 133.6–142.0°) [22]. This angle was found to be both significantly different/indifferent among the genders in the studied populations [1,17,26,32]. The angle of the head position was used for the estimation of the lower profile orientation [17] and, after the statistical analysis, no significant differences were found among the groups (Table 2). A wider angle points to a prognathic face profile, while narrower angles are found in those with retrognathic profiles. In male and younger female children, this angle was more acute than in other groups, indicating their retrognathic or posteriorly-divergent position of the lower face profile (Table 2).

The middle and lower thirds of the face height were assessed using the middle and lower facial third angles (Table 2). These two angles did not differ statistically among the groups, pointing to similar facial third sizes in all groups. However, when one compares these two angles between each other, it is visible that the angles of the lower facial third are much narrower than those of the middle facial third corresponding to the same group (Table 2). The only exception is the group of younger male children, where the lower facial third angle was wider than the middle facial third angle (Table 2), which can possibly be explained by the large SDs found for this angle. Similar findings as those of our study (i.e., that there are no sex or race differences) can be found in the literature [17].

## 5. Conclusions

This work represents the first photogrammetric analysis of facial soft-tissue in children and adults of the Serbian population. Besides the fact that this simple method is inexpensive, it can easily be repeated and gives important information concerning soft-tissue similarities between parents and their offspring. The authors are aware that the sample size might not be sufficient for explicit conclusions; however, this pioneering research can contribute to the pool of knowledge related to facial soft-tissue growth. Statistical analysis revealed the presence of significant differences among the groups of fathers/mothers and groups of male children aged 15–17, and female children aged 15–17 and 18–21 when the nasal and cervicomental angles, as well as the angle of the total facial convexity, were compared. These data suggest that there are much more similarities between the facial soft-tissue angles of fathers and their male offspring. Furthermore, mothers tend to have statistically insignificant differences in angles sizes, compared to both male and female offspring.

## Figures and Tables

**Table 1:** Facial landmarks used for the determination of linear and angular parameters.

Facial Landmarks	
Glabella (G)	The most anterior point of the middle line of the forehead.
Nasion (N)	The point in the middle line located at the nasal root.
Pronasal (Prn)	The most prominent point of the tip of the nose.
Columella (Cm)	The most inferior and anterior point of the nose.
Subnasal (Sn)	The point where the upper lip joins the columella.
Labial superior (Ls)	The point that indicates the mucocutaneous limit of the upper lip.
Labial inferior (Li)	The point that indicates the mucocutaneous limit of the lower lip.
Supramental (Sm)	The deepest point of the inferior sub-labial concavity.
Pogonion (Pg)	The most anterior point of the chin.
Menton (Me)	The most inferior point of the inferior edge of the chin.
Tragus (Trg)	The most posterior point of the auricular tragus.

**Table 2:** Photogrammetric analysis of facial angles in parents and their offspring, as well as the statistical comparisons among the groups.

Measured Angle	Groups of Subjects	N	Mean	SE	95% CI		*p*-Value (ANOVA)	*p*-Value (Tukey’s Post-Hoc)
					Lower Bound	Upper Bound		
Nasofrontal angle (G-N-Prn)	Male children 15–17 y.	6	148.42	2.28	142.57	154.27		
	Male children 18–21 y.	5	150.93	2.76	143.26	158.60		
	Female children 15–17 y.	6	149.57	1.80	144.95	154.20		
	Female children 18–21 y.	5	147.03	1.26	143.54	150.53		
	Fathers	15	142.64	9.39	122.51	162.77	0.952 ^a^	
	Mothers	15	153.94	1.40	150.94	156.95	0.066 ^b^	
Nasal angle (Cm-Sn/N-Prn)	Male children 15–17 y.	6	78.10	5.97	62.75	93.46		
	Male children 18–21 y.	5	85.61	2.71	78.08	93.14		
	Female children 15–17 y.	6	86.86	1.58	82.81	90.92		0.038 *
	Female children 18–21 y.	5	80.33	2.95	72.15	88.51		
	Fathers	15	72.01	2.99	65.60	78.42	0.027 ^a^	
	Mothers	15	76.43	1.94	72.28	80.58	0.079 ^b^	
Vertical nasal angle (N-Prn/TV)	Male children15–17 y.	6	30.81	0.93	28.42	33.20		
	Male children 18–21 y.	5	30.73	1.13	27.59	33.87		
	Female children 15–17 y.	6	32.87	0.82	30.75	34.98		
	Female children 18–21 y.	5	29.80	0.91	27.27	32.33		
	Fathers	15	29.77	0.93	27.77	31.76	0.280 ^a^	
	Mothers	15	29.51	0.69	28.04	30.99	0.092 ^b^	
Nasal dorsum angle (N-Mn-Prn)	Male children 15–17 y.	6	169.40	1.97	164.33	174.47		
	Male children 18–21 y.	5	169.51	2.01	163.93	175.09		
	Female children 15–17 y.	6	167.48	1.90	162.60	172.36		
	Female children 18–21 y.	5	172.37	0.90	169.87	174.86		
	Fathers	15	171.99	1.32	169.17	174.81	0.266 ^a^	
	Mothers	15	171.68	1.35	168.78	174.58	0.335 ^b^	
Nasolabial angle (Cm-Sn-Ls)	Male children 15–17 y.	6	105.13	5.73	90.41	119.86		
	Male children 18–21 y.	5	120.16	3.20	111.26	129.06		
	Female children 15–17 y.	6	117.00	4.16	106.30	127.70		
	Female children 18–21 y.	5	111.83	5.26	97.22	126.45		
	Fathers	15	109.52	3.83	101.31	117.73	0.297 ^a^	
	Mothers	15	111.19	3.46	103.76	118.62	0.296 ^b^	
Mentolabial angle Li-Sm-Pg	Male children 15–17 y.	6	132.66	2.80	125.46	139.86		
	Male children 18–21 y.	5	116.99	7.26	96.83	137.16		
	Female children 15–17 y.	6	139.89	9.57	115.29	164.49		
	Female children 18–21 y.	5	135.45	4.10	124.06	146.84		
	Fathers	15	126.72	3.57	119.06	134.37	0.115 ^a^	
	Mothers	15	129.44	3.21	122.56	136.32	0.124 ^b^	
Cervicomental angle C-Me/G-Pg	Male children 15–17 y.	6	96.88	1.41	93.25	100.51		0.037 *
	Male children 18–21 y.	5	99.45	4.24	87.68	111.22		
	Female children 15–17 y.	6	92.15	4.16	81.46	102.84		0.002 ***
	Female children 18–21 y.	5	97.17	3.29	88.02	106.32		
	Fathers	15	110.02	2.61	104.42	115.62	0.001 ^a^	
	Mothers	15	105.03	2.70	99.24	110.82	0.060 ^b^	
Middle facial third angle N-Trg-Sn	Male children 15–17 y.	6	25.06	0.75	23.14	26.97		
	Male children 18–21 y.	5	29.28	3.37	19.93	38.63		
	Female children 15–17 y.	6	24.51	0.29	23.77	25.24		
	Female children 18–21 y.	5	25.62	1.11	22.55	28.69		
	Fathers	15	27.65	1.69	24.02	31.28	0.477 ^a^	
	Mothers	15	25.73	1.06	23.47	28.00	0.341 ^b^	
Lower facial third angle Sn-Trg-Me	Male children 15–17 y.	6	27.36	3.34	18.77	35.96		
	Male children 18–21 y.	5	19.08	1.81	14.07	24.10		
	Female children 15–17 y.	6	22.74	3.43	13.92	31.56		
	Female children 18–21 y.	5	19.77	0.74	17.72	21.82		
	Fathers	15	20.74	1.48	17.58	23.91	0.166 ^a^	
	Mothers	15	20.21	1.55	16.90	23.53	0.154 ^b^	
Angle of the head position (Sn-Sm/TH)	Male children 15–17 y.	6	72.41	2.12	66.95	77.87		
	Male children 18–21 y.	5	72.83	1.76	67.94	77.72		
	Female children 15–17 y.	6	72.20	3.80	62.44	81.96		
	Female children 18–21 y.	5	81.78	1.25	78.31	85.25		
	Fathers	15	77.74	1.87	73.73	81.74	0.067 ^a^	
	Mothers	15	79.34	2.61	73.75	84.93	0.109 ^b^	
Facial profile angle (G-Sn-Pg)	Male children 15–17 y.	6	166.60	0.85	164.42	168.78		
	Male children 18–21 y.	5	162.88	2.12	157.00	168.76		
	Female children 15–17 y.	6	160.73	2.08	155.38	166.08		
	Female children 18–21 y.	5	168.41	2.87	160.43	176.38		0.062
	Fathers	15	167.09	1.35	164.20	169.98	0.053 ^a^	
	Mothers	15	167.40	1.33	164.54	170.26	0.043 ^b^	
Total facial convexity (G-Prn-Pg)	Male children 15–17 y.	6	146.89	1.61	142.74	151.03		0.012 **/0.06
	Male children 18–21 y.	5	139.84	2.81	132.05	147.63		
	Female children 15–17 y.	6	142.20	1.52	138.29	146.11		
	Female children 18–21 y.	5	145.39	2.51	138.43	152.35		
	Fathers	15	138.87	1.20	136.29	141.44	0.009 ^a^	
	Mothers	15	140.23	1.28	137.49	142.97	0.043 ^b^	

^a^ and ^b^—the statistical difference between groups of children, on one side, and fathers/mothers, on the other, respectively (ANOVA); * *p* < 0.05; ** *p* < 0.01, and *** *p* < 0.001 for statistically significant values after Tukey’s post-hoc test; SE – standard error; CI – confidence interval.

## References

[1] Anić-Milošević S., Lapter-Varga M., Slaj M. (2008). Analysis of the soft tissue facial profile by means of angular measurements. Eur. J. Orthod..

[2] Lapter Varga M., Anić-Milošević S., Vusić A., Slaj M., Varga S., Perinić M., Slaj M. (2008). Soft tissue facial profile of normal dental and skeletal subjects in Croatian population aged 12 to 15 years. Coll. Antropol..

[3] Bergman R.T., Waschak J., Borzabadi-Farahani A., Murphy N.C. (2014). Longitudinal study of cephalometric soft tissue profile traits between the ages of 6 and 18 years. Angl. Orthod..

[4] Cicciù M. (2017). Real opportunity for the present and a forward step for the future of bone tissue engineering. J. Craniofac. Surg..

[5] Mici E., Calvo A., Cicciù M., Cervino G., Belli E. (2018). Complex Orbital Fractures: Three-Dimensional Planning and Combined Surgical Approach. J. Craniofac. Surg..

[6] Cervino G., Fiorillo L., Arzukanyan A.V., Spagnuolo G., Cicciù M. (2019). Dental Restorative Digital Workflow: Digital Smile Design from Aesthetic to Function. Dent. J..

[7] Beugre J.B., Diomande M., Assi A.R., Koueita M.K., Vaysse F. (2017). Angular photogrammetric analysis and evaluation of facial esthetics of young Ivorians with normal dental occlusion. Int. Orthod..

[8] Bhandari V., Singla A., Mahajan V., Jaj H.S., Saini S.S. (2015). Soft tissue facial profile in Himachal population: A photogrammetric analysis. Indian J. Dent. Res..

[9] Djordjevic J., Zhurov A.I., Richmond S., Visigen Consortium (2016). Genetic and Environmental Contributions to Facial Morphological Variation: A 3D Population-Based Twin Study. PLoS ONE.

[10] Pandian K.S., Krishnan S., Kumar S.A. (2018). Angular photogrammetric analysis of the soft-tissue facial profile of Indian adults. Indian J. Dent. Res..

[11] Wen Y.F., Wong H.M., McGrath C.P. (2015). Longitudinal Photogrammetric Analysis of Soft Tissue Facial Changes: A Systematic Review of the Literature and a Summary of Recommendations. J. Craniofac. Surg..

[12] Vanco C., Kasai K., Sergi R., Richards L.C., Townsend G.C. (1995). Genetic and environmental influences on facial profile. Aust. Dent. J..

[13] King L., Harris E.F., Tolley E.A. (1993). Heritability of cephalometric and occlusal variables as assessed from siblings with overt malocclusions. Am. J. Orthod. Dentofac. Orthop..

[14] Lauweryns I., Carels C., Vlietinck R. (1993). The use of twins in dentofacial genetic research. Am. J. Orthod. Dentofac. Orthop..

[15] Hartsfield J.K., Morford L.A., Otero L.M., Bourzgui F. (2012). Genetic factors affecting facial growth. Orthodontics-Basic Aspects and Clinical Considerations.

[16] Aksakalli S., Demir A. (2013). The comparison of facial estethics between orthodontically treated patients and their parents. Sci. World J..

[17] Fernández-Riveiro P., Smyth-Chamosa E., Suárez-Quintanilla D., Suárez-Cunqueiro M. (2003). Angular photogrammetric analysis of the soft tissue facial profile. Eur. J. Orthod..

[18] Hoffelder L.B., de Lima E.M., Martinelli F.L., Bolognese A.M. (2007). Soft-tissue changes during facial growth in skeletal Class II individuals. Am. J. Orthod. Dentofac. Orthop..

[19] Subtelny J.D. (1961). The soft tissue profile, growth and treatment changes. Angl. Orthod..

[20] Ferrario V.F., Sforza C., Poggio C.E., Schmitz J.H. (1997). Three-dimensional study of growth and development of the nose. Cleft Palate Craniofac. J..

[21] Ferrario V.F., Sforza C., Poggio C.E., Schmitz J.H. (1999). Soft-tissue facial morphometry from 6 years to adulthood: A three-dimensional growth study using a new modeling. Plast. Reconstr. Surg..

[22] Wen Y.F., Wong H.M., Lin R., Yin G., McGrath C. (2015). Inter-Ethnic/Racial Facial Variations: A Systematic Review and Bayesian Meta-Analysis of Photogrammetric Studies. PLoS ONE.

[23] Prendiville S., Kokoska M.S., Hollenbeak C.S., Caplin D.A., Cooper M.H., Branham G., Thomas J.R. (2002). A comparative study of surgical techniques on the cervicomental angle in human cadavers. Arch. Fac. Plast. Surg..

[24] Malkoç S., Demir A., Uysal T., Canbuldu N. (2009). Angular photogrammetric analysis of the soft tissue facial profile of Turkish adults. Eur. J. Orthod..

[25] Asghari A., Rajaeih S., Hassannia F., Tavakolifard N., Fattahi Neisyani H., Kamrava S.K., Jalessi M., Omidian P. (2014). Photographic facial soft tissue analysis of healthy Iranian young adults: Anthropometric and angular measurements. Med. J. Islam. Repub. Iran.

[26] Wamalwa P., Amisi S.K., Wang Y., Chen S. (2011). Angular photogrammetric comparison of the soft-tissue facial profile of Kenyans and Chinese. J. Craniofac. Surg..

[27] Ajami S., Najafi H.Z., Mahdavi S. (2015). Angular photogrammetric analysis of the soft tissue facial profile of Iranian young adults. Iran. J. Orthod..

[28] Zylinski C.G., Nanda R.S., Kapila S. (1992). Analysis of soft tissue facial profile in white males. Am. J. Orthod. Dentofac. Orthop..

[29] Dimaggio F.R., Ciusa V., Sforza C., Ferrario V.F. (2007). Photographic soft-tissue profile analysis in children at 6 years of age. Am. J. Orthod. Dentofac. Orthop..

[30] Nanda R.S., Meng H., Kapila S., Goorhuis J. (1990). Growth changes in the soft tissue facial profile. Angl. Orthod..

[31] Lines P.A., Lines R.R., Lines C.A. (1978). Profilemetrics and facial esthetics. Am. J. Orthod..

[32] Epker B.N., McNamara J.A., Carlson D.S., Ferrara A. (1992). Adjunctive aesthetic surgery in the orthognathic surgery patient. Aesthetics and the Treatment of Facial Form.

